# [Corrigendum] miR‑216a‑5p acts as an oncogene in renal cell carcinoma

**DOI:** 10.3892/etm.2024.12443

**Published:** 2024-02-21

**Authors:** Peijie Chen, Jing Quan, Lu Jin, Canbin Lin, Weijie Xu, Jinling Xu, Xin Guan, Zebo Chen, Liangchao Ni, Shangqi Yang, Yun Chen, Yongqing Lai

Exp Ther Med 15:4039–4046, 2018; DOI: 10.3892/etm.2018.5881

Subsequently to the publication of the above article, the authors drew to the attention of the Editor that Figs. 5 and 6 both contained errors that arose inadvertently during the assembly of these figures. With the scratch wound assay data shown in Fig. 5A on p. 4043, the data panels showing the results from the ‘786-O/NC’ and ‘786-O/miR-216a inhibitor’ experiments at t=0 h were overlapping, and similarly, the’786-O/Migration, miR-216a mimic’ and ‘786-O/Migration, NC’ panels in Fig. 6A on p. 4044, showing the results of Transwell migration assay experiments, were overlapping, suggesting that these data were derived from the same original sources. After having consulted their original data, the authors realized where the errors occurred in assembling these figures, and were able to present the correct data for these figures to the Editorial Office.

New versions of Figs. 5 and 6, showing the correct data for the ‘786-O/miR-216a inhibitor’ experiment at t=0 h and the ‘786-O/NC’ experiment in Figs. 5A and 6A respectively) are shown on the next page. Note that the revised data shown for these figures do not affect the overall conclusions reported in the paper. All the authors agree with the publication of this corrigendum; furthermore, they apologize to the Editor of *Experimental and Therapeutic Medicine* and to the readership for any inconvenience caused.

## Figures and Tables

**Figure 5:**
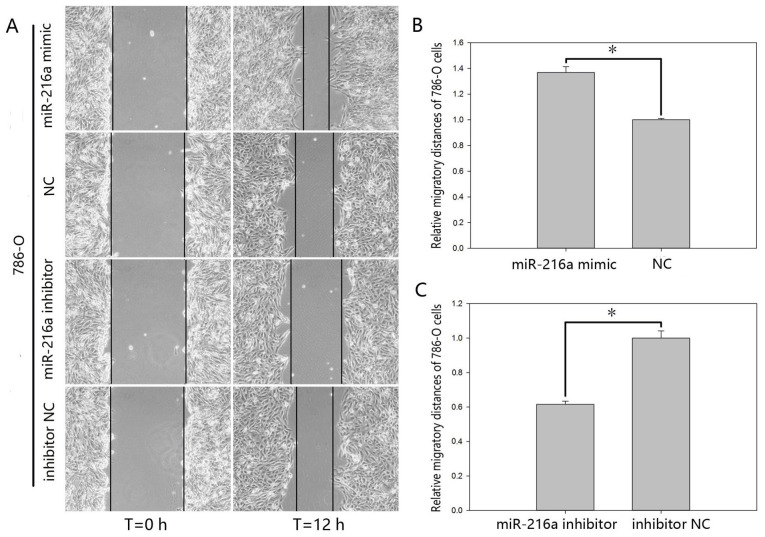
Wound scratch assay of 786-O cells. (A) Migratory images captured of cells. Data of migratory distances in 786-O cells transfected with (B) miR-216a-5p mimic or NC and (C) miR-216a-5p inhibitor or inhibitor NC. ^*^P<0.05. miR, microRNA; NC, negative control.

**Figure 6:**
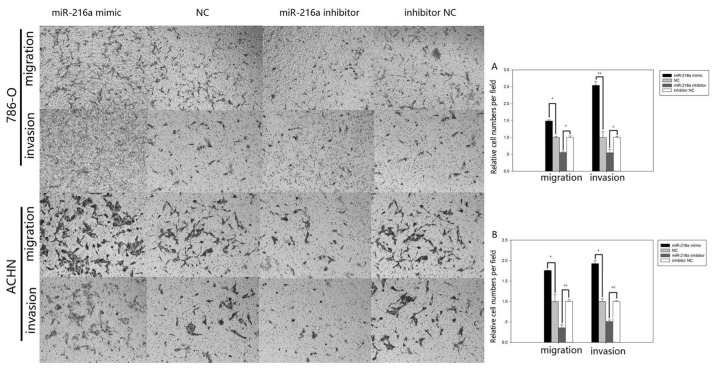
Transwell assay in 786-O and ACHN cells. (A) Migratory and invasive images captured of 786-O and ACHN cells. The results of migration and invasion in (B) 786-O and (C) ACHN cells transfected with miR-216a-5p mimic or NC and miR-216a-5p inhibitor or inhibitor NC. *P<0.05, **P<0.01. miR, microRNA; NC, negative control.

